# Persistence of microbial extracellular enzymes in soils under different temperatures and water availabilities

**DOI:** 10.1002/ece3.6677

**Published:** 2020-08-17

**Authors:** Enrique J. Gómez, Jose A. Delgado, Juan M. González

**Affiliations:** ^1^ Instituto de Recursos Naturales y Agrobiología de Sevilla (IRNAS) Consejo Superior de Investigaciones Científicas (CSIC) Sevilla Spain

**Keywords:** extracellular enzyme activity, organic matter decomposition, persistence, soils, temperature, water activity, water availability

## Abstract

Microbial extracellular enzyme activity (EEA) is critical for the decomposition of organic matter in soils. Generally, EEA represents the limiting step governing soil organic matter mineralization. The high complexity of soil microbial communities and the heterogeneity of soils suggest potentially complex interactions between microorganisms (and their extracellular enzymes), organic matter, and physicochemical factors. Previous studies have reported the existence of maximum soil EEA at high temperatures although microorganisms thriving at high temperature represent a minority of soil microbial communities. To solve this paradox, we attempt to evaluate if soil extracellular enzymes from thermophiles could accumulate in soils. Methodology at this respect is scarce and an adapted protocol is proposed. Herein, the approach is to analyze the persistence of soil microbial extracellular enzymes at different temperatures and under a broad range of water availability. Results suggest that soil high‐temperature EEA presented longer persistence than enzymes with optimum activity at moderate temperature. Water availability influenced enzyme persistence, generally preserving for longer time the extracellular enzymes. These results suggest that high‐temperature extracellular enzymes could be naturally accumulated in soils. Thus, soils could contain a reservoir of enzymes allowing a quick response by soil microorganisms to changing conditions. This study suggests the existence of novel mechanisms of interaction among microorganisms, their enzymes and the soil environment with relevance at local and global levels.

## Graphical abstract

Soil microbial extracellular enzyme activity is highest at high temperature even if thermophiles represent a minimum fraction of the total bacterial community. We study the persistence of mesophilic and thermophilic enzymes in soils in relationship to temperature and water content. Enzymes active at high temperature (i.e., 60°C) last longer time than those from mesophiles. High‐temperature extracellular enzymes can accumulate in soils which explains the high extracellular enzyme activity at high temperatures observed in soils. This study contributes to better understand microbe‐organic matter interactions in soils and predicts a significant influence of global warming on soil microbial extracellular enzyme activity.

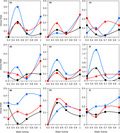

## 1 INTRODUCTION

Microorganisms govern the functioning of soil biogeochemical cycles (Conant et al., 2011; Whitman, Coleman, & Wiebe, 1998). The mineralization of soil organic matter to CO_2_ is relevant on climate warming because of its effect on the role of soils as sink or source of C (Davidson & Janssens, 2006; IPCC, 2014). Understanding how soil organic matter is being processed by microorganisms is essential for the knowledgeable management of soils and modeling of global climate scenarios.

Soils contain a high proportion of organic matter resulting, for instance, from plant residues. According to the biodegradability of soil organic matter, different reports (Conant et al., 2011; Guimarães et al., 2013; Wallenstein & Burns, 2011) suggest different rates of consumption of distinct fractions. Some of these fractions are constituted by recalcitrant compounds which present long‐lasting permanence in soils (Cheng et al., 2017; Conant et al., 2011; Guimarães et al., 2013). Temperature is a major factor ruling the dissolution of some soil organic compounds (Allison & Treseder, 2008; Biederman et al., 2016; Cheng et al., 2017; Conant et al., 2011) increasing the range of available organic compounds at increasing soil temperature. As well, soil texture and water availability can greatly influence the biodecomposition rate of some organics in soils (Borowik & Wyszkowska, 2016), for instance, because of the organic matter that complexes with clay particles (Datta et al., 2017) and the adhesion of organic matter to soil particles during desiccation or drought periods (Datta et al., 2017; Hammerl et al., 2019). Thus, the environment affects the composition and availability of soil organic matter to microorganisms.

The first step to decompose high‐molecular weight organic matter by microorganisms consists in the use of extracellular enzymes. These extracellular enzymes assist in breaking down large molecules or polymers into much smaller subunits and monomers which can be incorporated into the microbial cells to produce biomass and to obtain energy. The extracellular enzyme activity (EEA) is generally the limiting step in the process of biodecomposition and mineralization of soil organic matter (Cheng et al., 2017; Conant et al., 2011; Gonzalez, Portillo, & Piñeiro‐Vidal, 2015). Thus, the study of factors and mechanisms involved on EEA in soils is of decisive importance to understand soil organic matter bioprocessing.

Extracellular enzyme activity has been measured for many years at a single temperature, generally at or below 30°C, in an aqueous solution (Fierer, Colman, Schimel, & Jackson, 2006; Townsend, Vitousek, & Holland, 1992). Nevertheless, soil upper layers frequently get dry and experience high temperatures. These extreme conditions present a common scenario in many soils, above all, those classified as semiarid, arid, and deserts (Hammerl et al., 2019). The functional responses of soil microorganisms, microbial processes, and specifically their extracellular enzymes to changing conditions remain to be understood (Jian et al.., 2016; Xiao, Chen, Jing, & Zhu, 2018).

Today, it is known that thermophilic microorganisms are ubiquitous in soils (Marchant, Banat, Rahman, & Berzano, 2002; Portillo, Santana, & Gonzalez, 2012; Santana & Gonzalez, 2015). A potentially important role for soil thermophiles has been shown to recycle organic C, N, and S (Portillo et al., 2012; Santana & Gonzalez, 2015; Santana, Portillo, González, & Clara, 2013). Gonzalez et al. (2015) demonstrated the existence of peaks of maximum EEA at high temperatures in all soils tested from different latitudes. This suggested that the elevated high‐temperature EEA might be a consequence of the activity being carried out by soil thermophilic microorganisms. This finding has suggested (Santana & Gonzalez, 2015; Aksoy, Yigini, & Montanarella, 2016) the potential of soil thermophiles to be responsible for a decrease of soil organic matter content in soils exposed to high temperatures.

In spite of the existing peaks of maximum EEA at high temperature observed in soils, thermophilic microorganisms only represent a minimum fraction of soil microbial communities (Portillo et al., 2012). Consequently, that reduced fraction of soil thermophiles could not easily explain the high‐temperature EEA measured in soils. Two potential causes for that high soil EEA at elevated temperatures could be proposed. On one hand, soil thermophiles could present high activity during high‐temperature periods. Although the number of days presenting high temperatures can represent a highly significant fraction of the year at medium to low latitudes (i.e., about 1/3 of the year in Seville, Spain) (Gonzalez et al., 2015), the high rates of extracellular enzyme production by thermophiles required to account for those EEA measured in soils do not look feasible for this option to be realistic. On the other hand, the extracellular enzymes produced by soil thermophiles during high‐temperature events could accumulate in the soil environment and so a high stock of high‐temperature extracellular enzymes could be readily available. This option would be supported by the higher stability and resistance reported for enzymes from thermophiles than from their mesophilic homologues (Vieille & Zeikus, 2000).

The persistence of enzymes in soils has been barely studied. Renella, Szukics, Landi, and Nannipieri (2007) presented a procedure to determine enzyme production and persistence in soils. Generally, enzyme production by mesophilic microorganisms was higher that their persistence and these rates appeared to be unrelated to soil pH (Renella et al., 2007). Although there are many aspects of EEA and its persistence in soils that remain to be studied, that study (Renella et al., 2007) showed a first methodological approach to estimate of the production and persistence of hydrolythic enzymes in soils.

This study aims to comparatively determine the possibility of high persistence of extracellular enzymes from mesophilic and thermophilic microorganisms in soils as a potential explanation for the high‐temperature EEA in soils (Gonzalez et al., 2015). This will be performed under different conditions of temperature and water availability which represent distinct scenarios typically observed in soils but rare approached. Results could lead to potential consequences on the responses and strategies of microorganisms to soil organic matter with relevance at local and global scales, above all, under the current climate change scenario.

## 2 MATERIALS AND METHODS

### 2.1 Sampling sites

Upper layer soil samples were collected from three different locations at the Iberian Peninsula (Table 1). These samples correspond to a silt soil (Benasque, Huesca, Northern Spain; 42°40.922′N 000°38.108′E), a sandy loam soil (Coria del Rio, Sevilla, Southern Spain; 37°17.027′N, 006°3.973′W), and a sandy clay loam soil (Tavizna, Cadiz, Southern Spain; N 36°46.687′N, 005°29.557′W).

**Table 1:** Characteristics of the soils sampled in this study. Sampling sites are organized in the table from North (upper) to South (lower)

	Location	Coordenates	Soil type (texture)	Temp. (°C)^a^	Precip. (mm)^a^	Koppen‐Geiger Clymate Type
Northeastern Spain	Benasque, Huesca	N 42°40.922′ E 000°38.108′	Silt	8.2	1,013	Cfb
Southwestern Spain	Coria del Río, Sevilla	N 37°17.027′ W 006°3.973′	Sandy loam	18.4	572	Csa
Southwestern Spain	Tavizna, Cádiz	N 36°46.687′ W 005°29.557′	Sandy clay loam	17.6	739	Csa

^a^ Values of Temperature (Temp.) and Precipitation (Precip.) correspond to annual means.

### 2.2 Water availability measurements

Water availability was quantified by water activity (a_w_; from 1, water saturated, to 0, no water). Water activity represents the quotient of the water vapor pressure for the sample against the vapour pressure in distilled water under the same conditions. The response of microorganisms to water scarcity was evaluated at different water activity values (Grant, 2004; Stevenson et al., 2015). The required water activity in soil samples was obtained by partial drying in a vacuum concentrator at room temperature. Different drying time periods result in different levels of water activity. Longer drying times resulted in lower water activity. Water activity of soil subsamples was measured using a Rotronic water activity probe HC2‐AW (Rotronic AG).

### 2.3 Decay curves under different temperature and water availability conditions

Samples at the desired water activity were incubated in triplicate in sealed bottles so that the water activity of the samples was maintained constant during incubation. Incubations and enzyme assays were carried out at two temperatures, 20°C and 60°C, and under four different water activity conditions (a_w_ 0.3, 0.5, 0.7, and 1). Subsamples were preincubated for 24 hr at 20°C and 60°C to select the enzymes working optimally at these temperatures and to reach a maximum yield of these enzymes during this period of enzyme production. Unlike the procedure by Renella et al. (2007), herein, samples remained unsupplemented. During this preincubation, most available nutrients are consumed and so the production of enzyme during the decay phase of the experiment will be minimized. After the preincubation, the net EEA initiated a decrease over time (Renella et al., 2007). Equal portions of the samples preincubated at 20°C and 60°C were used to initiate the decay curves by incubation at those temperatures. Aliquots were collected at 0, 1, 2, 3, and 4 hr of incubation. Aliquots were immediately stored at −20°C until processed.

#### 2.3.1 Enzyme assays

Enzyme assays to determine EEA along the decay curve were performed in triplicate. Each replicate contained 2 mg of soil from an aliquot collected at a time period as mentioned above. The reaction mixtures were supplemented with buffer, either phosphate buffer (0.2 M, pH 7) for protease and glucosidase assays or PIPES buffer (2 mM, pH 7) for phosphatase assays, and a fluorogenic substrate analogue (0.1 mM, final concentration; Gomez, Delgado, & Gonzalez, 2020; Gonzalez et al., 2015) specific for each enzyme to be assayed. The replicate mixtures were maintained on ice until a fluorogenic substrate was added. The fluorogenic substrates were L‐leucine‐7‐amido‐4‐methylcoumarinhydrochloride (AMC) for protease activity, Methylumbelliferil‐β‐glucopyranoside (MUG) for glucosidase activity, and Methylumbelliferil‐phosphate (MUP) for phosphatase activity.

Enzyme assays to determine the activity of enzymes from thermophilic microorganisms were performed at 60°C and assays to determine the activity of enzymes from mesophilic microorganisms were incubated at 20°C. Incubations up to 5 min were carried out and aliquots were collected over time to determine EEA rates. The enzyme assay reactions were stopped by adding ice‐cold glycine‐NaOH (0.1 M, pH 11) so that the final pH of the solution maximized the fluorescent signal (Stemmer, 2004). Fluorescent measurements were performed using an Omega fluorometer (BMG LabTech GmbH, Ortenberg, Germany) with the filter sets recommended by the manufacturer. Substrate thermal stability has been tested to ensure the appropriateness of these substrates at the assay temperatures, for example, testing the lack of substrate cleavage during assays in the absence of the corresponding enzyme (Gonzalez et al., 2015). Regression analysis (Sokal & Rohlf, 2012) of the assay curve was carried out to estimate EEA rate using the linear portion of the fluorescence versus time (in min) curve.

### 2.4 Analysis of the decay curve results

Enzyme persistence was estimated from the decay curve of the EEA rate versus incubation time (in days). High decay rates correspond to low persistence and vice versa, low decay rates imply long persistence. Persistence was inverse to the decay rate of EEA versus time. This model was based on the procedure proposed by Renella et al. (2007) with some modifications. Our procedure did not supplement the samples with nutrients previous to the preincubation. Herein, the proposed procedure incorporated the estimation of the decay rate of enzymes as a first‐order kinetics from the linear relationship obtained when plotting the natural logarithm of EEA rate versus time (in days) for each enzyme and condition of incubation. The estimated decay rates actually represent net decay rates resulting from the decrease of EEA and a minor fraction of enzyme being produced during the decay portion of these experiments. Decay of EEA from thermophiles at 20°C is assumed to only account for the slow inactivation of thermophilic enzymes in soils over time because the metabolism of thermophiles is assumed to be inhibited at this temperature. Decay of EEA from mesophiles at 60°C is assumed to represent mainly the result of thermal inactivation of enzymes from mesophilic microorganisms in soils during high‐temperature events. It is assumed that no overlap of EEA from thermophilic and mesophilic microorganisms exists at 60°C and 20°C as previously shown by Gonzalez et al. (2015).

### 2.5 Multivariate analysis

The response of decay rates for different enzymes and for those enzymes under different temperature and water availability conditions in various soils was analyzed by a multi‐response permutation procedure (MRPP) using Bray dissimilarity distances in R (Warton, Wright, & Wang, 2012). Visualization of the ordination of decay results for different enzymes, temperatures, water availabilities, and soils was achieved by nonmetric multidimensional scaling (NMDS) analysis using the vegan package in R (Oksanen et al., 2011).

## 3 RESULTS

### 3.1 Preliminary results

**Figure 1:**
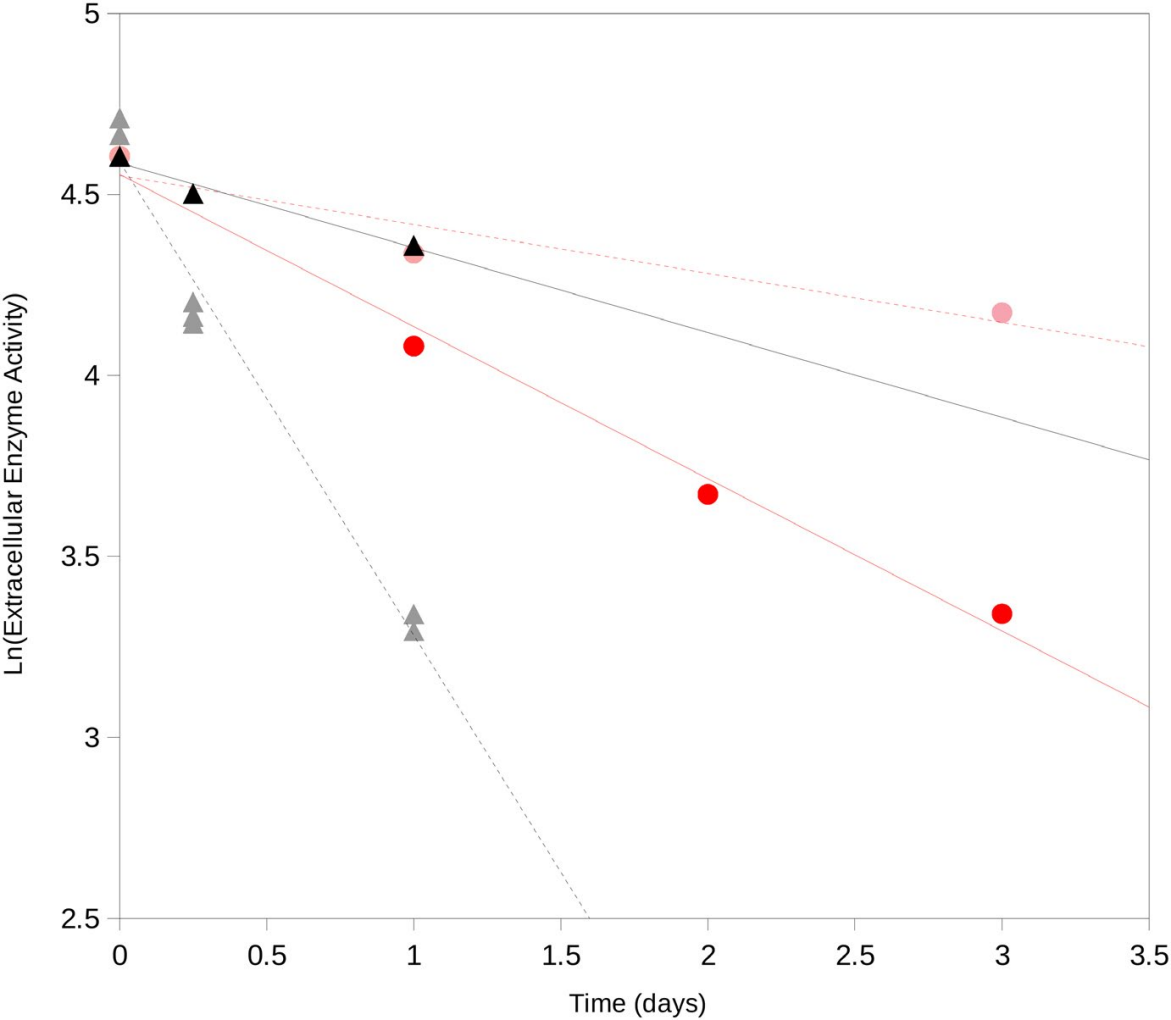
Examples of the decay curves for extracellular enzymes from mesophiles and thermophiles at 20°C and 60°C in a South Spain (Seville) soil under water activity 1 (wet conditions). Red circles, decay of enzyme activity from thermophiles at 60°C; pink circles, decay of enzyme activity from thermophiles at 20°C; black triangles, decay of enzyme activity from mesophiles at 20°C; gray triangles, decay of enzyme activity from mesophiles at 60°C. Points are average values from triplicates. Error bars indicate a standard deviation

Preliminary results showed that EEA decay curves followed a first‐order kinetics (Figure 1). The proposed procedure allowed the estimation of decay constant rates as a linear relationship of the natural logarithm of the EEA rate over incubation time. As a result, the length of the decay experiments can be reduced from over a month (Renella et al., 2007) down to around 4 hr. Thus, the improvement greatly facilitates enzyme decay rate estimates in soil samples simplifying the mathematics and experimental procedure proposed by Renella et al. (2007).

### 3.2 Decay curves under soggy conditions (a_w_ 1)

Figure 1 shows an example of the decay curve for the EEA of protease from mesophilic and thermophilic microorganisms at 20°C and 60°C. in a soil from Seville (South Spain) at a value of water activity 1 (i.e., wet conditions). This figure presents a comparison of the decay of enzymes from mesophilic microorganisms exposed at 20°C and 60°C and their comparisons with the EEA from thermophilic microorganisms exposed at 20°C and 60°C. Results showed that EEAs from mesophilic microorganisms present a good persistence (low decay rate) at 20°C but it is rapidly inhibited at 60°C. EEA from thermophilic microorganisms presented long persistence (very low decay rate) at 20°C and a much lower slope (decay rate) than their mesophilic counterparts at high temperature (60°C). A microphotograph of an example of a typical soil thermophilic bacterial isolate has been recently shown (Figure 1 in Gomez et al., 2020).

**Figure 2:**
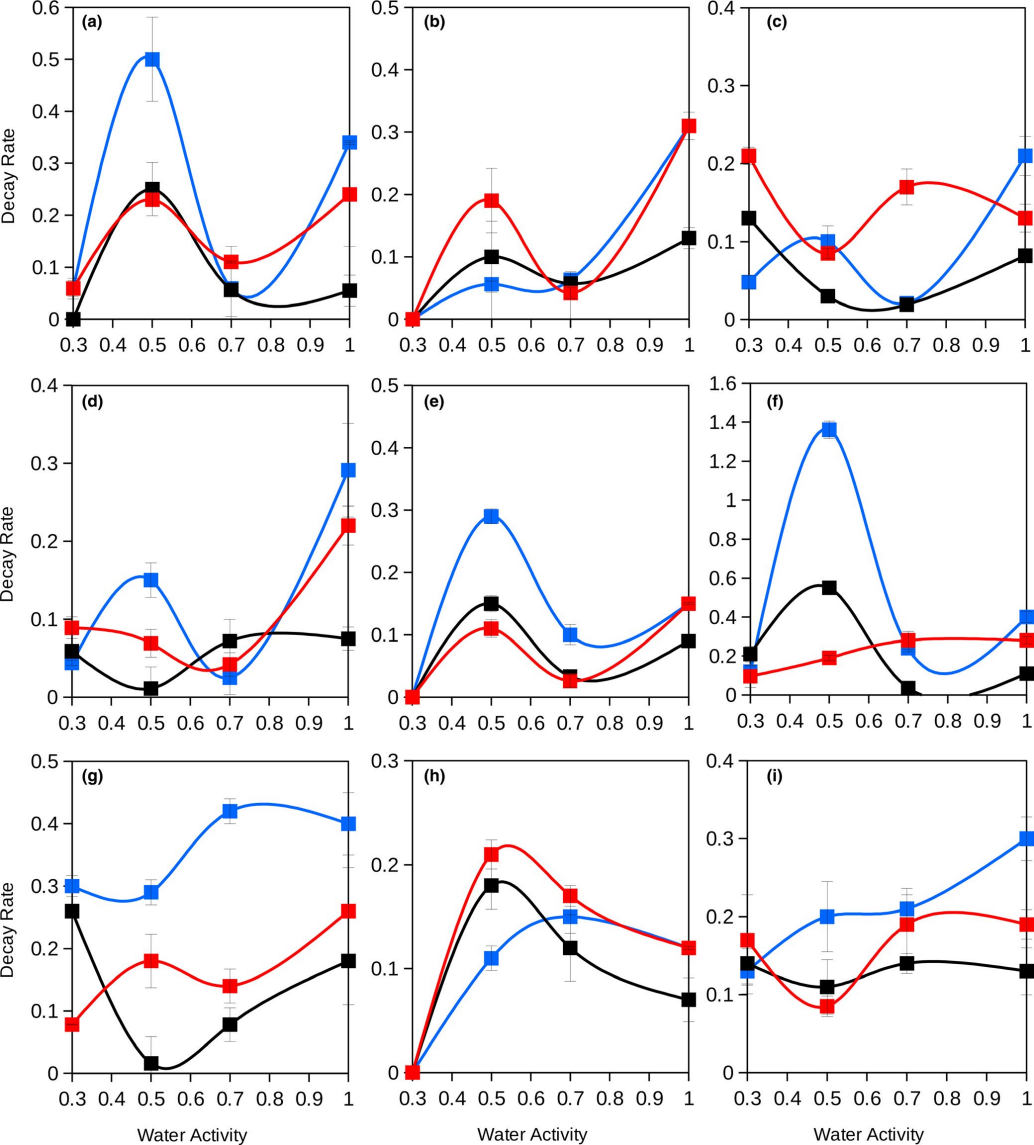
Decay rates as a function of water availability and temperature for extracellular enzymes from mesophiles and thermophiles at three different soils. Extracellular enzymes: a, b, and c (upper row), glucosidases; d, e, and f (central row), phosphatases; g, h, and i (lower row), proteases. Left column (a, d, and g), Seville soil (South Spain); Center column (b, e, and h), Cadiz soil (South Spain); Right column (c, f, and i), North Spain soil. Symbols: in red, decay of extracellular enzymes from thermophiles at 60°C; in blue, decay of extracellular enzymes from mesophiles at 20°C; in black, decay of extracellular enzymes from thermophiles at 20°C. Points resulted from the average of triplicated samples. Error bars indicate a standard deviation

At high water activity (a_w_ 1; Figure 2), results showed that enzymes from thermophilic microorganisms presented a higher persistence (lower decay rate) than EEA from mesophilic microorganisms. Significant differences were observed between the decay at these different temperatures and types of enzymes (*p* < .007. MRPP analysis).

### 3.3 Decay curves under different water activity conditions

Besides the effect of temperature, EEA decay is also affected by water availability (Figure 2). At low a_w_ values (i.e., under dry conditions) an increased variability in the decay response of the enzymes was observed. Significant differences of decay rates were observed for glucosidase, phosphatase, and protease activities at different water activities (*p* < .005, .005, and .04, respectively. MRPP analysis). The response of the three tested EEAs was significantly different (*p* < .001. MRPP analysis). For example, at a_w_ 0.5 (which represent conditions below the potential growth of any microorganism; Stevenson et al., 2015) maximum decay rates were frequently observed for different enzymes in distinct soils indicating adverse conditions for both microorganisms and their extracellular enzymes (Figure 2). Most enzymes in different soils presented lowest decay rates at a_w_ 0.7 which represents quite dry conditions close to the lowest a_w_ limit for microbial growth. The lowest a_w_ value assayed (a_w_ 0.3; extreme dry soil conditions) presented very low decay rates as a consequence of very poor measurable EEA indicating that at these conditions a residual activity of microbial extracellular enzymes is maintained in soils.

**Figure 3:**
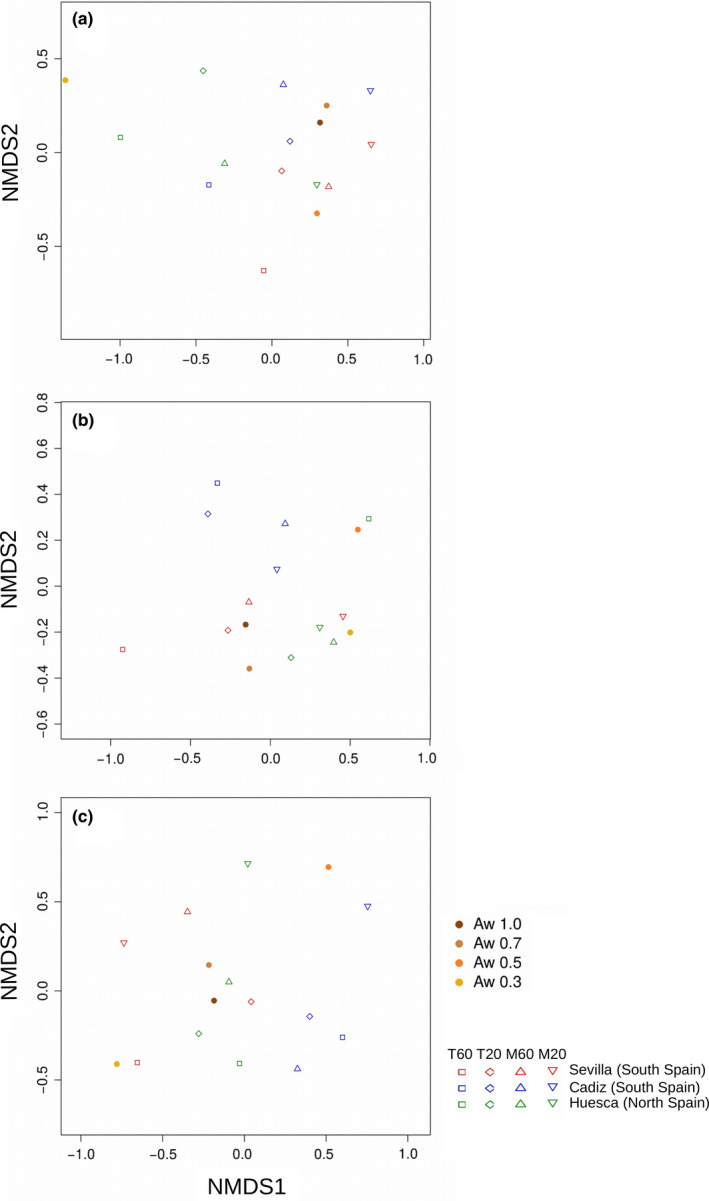
NMDS ordination of decay rates as a function of temperature, water availability and the soils for glucosidase (a), phosphatase (b), and protease (c) activities. Water activity is shown with brownish filled circles from dark to light in decreasing levels of water activity. Decays at 20°C and 60°C by enzymes from mesophilic (M20, down‐pointing triangles, and M60, up‐pointing triangles, respectively) and thermophilic (T20, squares, and T60, diamonds, respectively) microoorganisms are shown as unfilled symbols for each studied soil (Huesca soil [North Spain] in green, Cadiz soil [South Spain] in blue, Seville soil [South Spain] in red)

Ordination by NMDS analysis of the different parameters tested (i.e., temperature, water availability, and soils) for the decay of studied EEAs confirmed increased differentiation on decay rates when reducing water availability (Figure 3). In most cases, decay rates at a_w_ 1 and a_w_ 0.7 were located in proximity whereas the highest divergence was observed for a_w_ 0.5 and a_w_ 0.3 (the most dried conditions). NMDS stress values for glucosidase, phosphatase, and protease activities were 0.07, 0.06, and 0.04, respectively, suggesting excellent representations of decay rates observed for the tested enzymes under the variety of analyzed conditions. The decay at 60°C of enzymes from thermophiles showed minimum distance among the three EEAs tested. Generally, the most dispersed decays between the different soils were observed for EEA decay at 20°C for mesophilic and thermophilic microorganisms. These results suggested an homogeneous behavior of high‐temperature enzymes under elevated temperatures showing high persistence. At moderate temperature (20°C), the results for EEA decay from mesophilic and thermophilic microorganisms showed different responses.

## 4 DISCUSSION

Extracellular enzymes represent the limiting step for the decomposition of complex soil organic matter (Cheng et al., 2017; Conant et al., 2011; Gonzalez et al., 2015). Besides the relevance of EEA in soils and its consequences for the soil‐atmosphere C balance (Conant et al., 2011; Davidson & Janssens, 2006; IPCC, 2014), scarce information is available on the persistence of these enzymes and their implications for soil functioning depending on soil variables such as temperature and water content. Herein, we propose an improvement to a previously reported protocol to assess soil microbial EEA persistence and we present the first analysis of the decay of extracellular enzymes under a broad range of conditions determined by temperature and water availability. This study contributes to better understand the role of soil microbial extracellular enzymes under standard soil conditions including extreme temperature and desiccation events commonly observed in natural soils.

The observation that the EEA decay curve in soils follows a first‐order kinetics allows to facilitate decay rate estimates by linear regression of the natural logarithm of remaining EEA over time. Besides, this procedure avoids the previous ambiguity to quantify the decay rate over an asymptotic EEA decay curve (Renella et al., 2007) and greatly reduces the incubation time required to complete an EEA decay estimation from about 30 days (Renella et al., 2007) down to around 4 hr.

Enzymes in the soil environment are influenced by diverse factors. Soil microbial EEA is the result of a variety of multiple enzymes produced within a complex microbial community. The components of these communities are expected to release extracellular enzymes which can perform optimally under different conditions. The existence of distinguishable sets of extracellular enzymes suggests a potential distinctive role of these types of enzymes filling up different niches in the ecosystem. As an example, we observed the presence of extracellular enzymes working at moderate temperature and others optimized for 0.1–0.5 day^−1^ high temperature. Gonzalez et al. (2015) confirmed the presence of a major component of the whole soil EEA performing under high temperature. Upper soil layers can frequently get hot (Gonzalez et al., 2015; Portillo et al., 2012; Santana & Gonzalez, 2015) and so these thermophilic enzymes could perform optimally in this scenario.

Soil contains a large fraction of complex organic matter whose decomposition can be significantly assisted by high‐temperature events (Cheng et al., 2017; Conant et al., 2011; Hammerl et al., 2019). Persistence of enzymes from soil thermophilic microorganisms (e.g., *Geobacillus*‐related bacteria; Santana & Gonzalez, 2015) could suggest the existence of a soil reservoir of high‐temperature enzymes available to decompose polymers during high‐temperature events which are commonly observed in soil upper layers. Nevertheless, increasing soil temperature is paralleled to a decrease in soil water content (Lakshmi, Jackson, & Zehrfuhs, 2003). At this respect, the functioning of microbial extracellular enzymes in soils could be affected by this understudied scenario (Gomez et al., 2020; Moxley, Puerta‐Fernández, Gómez, & González, 2019). Herein, we present an approach to estimate the potential persistence of microbial EEA in soils under a variety of temperature and water availability conditions.

Soil water content significantly affects EEA (Gomez et al., 2020; Moxley et al., 2019). Depending on the level of water availability or grade of dryness microbial EEA can present high persistence and durability in the soil ecosystem (i.e., at a_w_ ≥ 0.7). At low water activity values (i.e., around a_w_ 0.5), high decay rates were observed suggesting a potential reduction of the persistence of enzymes under these conditions. Thus, water content, and specifically how dry is a soil, can present important consequences to evaluate the persistence of EEA in soils. Our results showed that the persistence of soil microbial EEA is highly dependent on water availability and temperature.

Results from this study showed that most estimates of decay rates for different EEAs under a variety of conditions ranged 0.1–0.5 day^−1^. These estimates were obtained in unsupplemented soils which were analyzed with minimum alterations to their natural state. A handicap of using unsupplemented soil samples with the proposed methodology is that the extracellular enzyme production rates cannot be estimated. This is because nutrients and substrate availability limit enzyme synthesis (Burns et al., 2013). A benefit of the proposed procedure is the potential to obtain realistic estimates of actual decay rates for soil EEAs under a variety of temperature and water availability conditions which significantly influence the rate of EEA decay in soils. Renella et al. (2007) presented results on hydrolase production and persistence after supplementing soil samples with nutrients at a constant 25°C temperature. Nutrient supplementation certainly increased the rates of production, and likely decay, of soil extracellular enzymes leading to overestimates of the actual rates in soils. The results by Renella et al. (2007) showed enzyme decay rates from 0.49 to 3.7 day^−1^ for proteolytic soil enzyme activity. The values of decay observed in our study were at the low range of those reported by Renella et al. (2007).

Microbial cells in soils rapidly respond to changes in their environment (Burns et al., 2013; Conant et al., 2011; Renella et al., 2007; Wallenstein & Weinstraub, 2008). Soil microorganisms adapted to new conditions should be able to quickly activate their cellular machinery, including activation of protein synthesis. As examples, we can highlight the reported rapid response offered by soil thermophiles to high soil temperature events (Portillo et al., 2012; Santana & Gonzalez, 2015) and the very quick responses by soil microorganisms to rewetting events (Allison & Treseder, 2008; Conant et al., 2011; Jian et al., 2016; Xiao et al., 2018). These cases suggest that soil microorganisms should be able to respond to common environmental changes within a short time frame, well below an hour (Burns et al., 2013; Conant et al., 2011; Renella et al., 2007; Wallenstein & Weinstraub, 2008). Our results suggest low extracellular enzyme decay rates (i.e., <0.2 day^−1^) in soils indicating long persistence of these enzymes. The long persistence of enzymes and the fast response by microorganisms suggests that microbial extracellular enzymes could accumulate in soils and potentially contribute to fast responses.

The durability of microbial enzymes in soil represents a critical factor to understand the processes of complex organic matter decomposition in soils. This first step in the processing of soil organic matter by microorganisms does not necessarily need to be accompanied by microbial growth (Gomez et al., 2020). The existence of a stock of active extracellular enzymes could start organic matter processing (i.e., hydrolysis) even before or after microbial growth occurs (activation and inhibition periods of growth, respectively). For example, this implies dry and high‐temperature conditions limiting the growth of mesophilic microorganisms could activate high‐temperature EEA and accelerate the release of monomers and small organics even before specific cells (i.e., soil thermophiles) are able to start cell division. This would facilitate nutrient assimilation by viable cells during their early lag phase of growth. This represents a novel concept for organic matter processing in soils which would explain fast microbial responses to changes in soil organics and environmental parameters (e.g., organic nutrients, temperature, and water availability). The above suggests a singular adaptive mechanism to rapidly and efficiently process available organic nutrients by soil microorganisms in a changing environment.

The large fraction of organic matter in soils constitutes a reservoir of C with potentially singular consequences for global warming and the soil‐atmosphere C balance (Conant et al., 2011; Davidson & Janssens, 2006; IPCC, 2014). Most organic matter is held at upper soil layers (López‐Bellido, Lal, Danneberger, & Street, 2010). Some of that organic matter presents increased solubility at high temperature (Harrison‐Kirk, Beare, Meenken, & Condron, 2014; Lakshmi et al., 2003) so that it only becomes accessible to microbial processing during high‐temperature events. As well, variations of temperature and water content (i.e., daily cycles of temperature and water content; Harrison‐Kirk et al., 2014; Lakshmi et al., 2003; Portillo et al., 2012) could significantly contribute to increase the concentration of organic compounds as a consequence of evaporation and water volume reduction (Cheng et al., 2017; Conant et al., 2011; Hammerl et al., 2019; Lakshmi et al., 2003). The activity of extracellular hydrolytic enzymes can be facilitated by increased polymeric substrate solubility and increased organic substrate concentration.

The existence of enzymes presenting differential characteristics in soil microbial communities offers a range of possibilities for improved performance on the processing of soil organic matter under highly different conditions. Above all, the persistence of EEA in soils could be a major factor governing microbial decomposition of resilient fractions of soil organic matter. The role of mesophilic and thermophilic microorganisms could be complementary and both could benefit of the presence of active EEA in soils. The case of soil thermophilic microorganisms is the simplest scenario to understand. For example, thermophiles are assumed to function during high‐temperature extreme events (i.e., at and above 40°C; Portillo et al., 2012) which are fairly common in soils at medium to low latitudes (Gonzalez et al., 2015). The availability of functional extracellular enzymes in soils would accelerate the incorporation of nutrients by these thermophilic cells and reduce the time needed to activate their metabolism leading to reductions of their lag phase of growth and to a speedy organic matter processing strategy in natural soils. This strategy justifies the benefits of the high‐temperature EEA observed in soils by Gonzalez et al. (2015).

Under the current global warming scenario, it is expected an increase of the frequency and intensity of high‐temperature events (Conant et al., 2011; Davidson & Janssens, 2006; IPCC, 2014) which should be reflected in an increased relevance of thermophilic processes in soils. The contribution by thermophilic processes should be considered in future global climate predictions.

## 5 CONCLUSION

Soil microbial EEA represents a decisive and limiting step in the processing of soil organic matter. The persistence of these enzymes and the characterization of the enzymes from soil microbial communities should contribute to better understand how and when these enzymes perform and their local and global consequences. EEA persistence in soils can result in significant feedback mechanisms to self‐maintain particular microbial communities in soils by allowing faster responses to environmental changes (i.e., temperature and water availability) by microorganisms, both at the mesophilic and thermophilic growth range of temperatures. Temperature and water availability show decisive influence on EEA and its persistence in soils. Extracellular enzymes, mainly those from thermophilic microorganisms, can accumulate in soils which represent a new strategy that significantly contributes to better understand soil functioning at a local scale (i.e., ecosystem), as well as to better predict potential future global warming scenarios.

## CONFLICT OF INTEREST

There are no conflicts of interest to declare.

## AUTHOR CONTRIBUTION

**Enrique Jose Gomez:** Conceptualization (supporting); Investigation (lead); Methodology (equal); Writing‐review & editing (supporting). **Jose Antonio Delgado:** Conceptualization (supporting); Investigation (equal); Methodology (equal); Writing‐review & editing (supporting). **Juan Miguel Gonzalez:** Conceptualization (lead); Data curation (lead); Formal analysis (lead); Funding acquisition (lead); Investigation (equal); Methodology (equal); Project administration (lead); Resources (lead); Supervision (lead); Writing‐original draft (lead); Writing‐review & editing (lead).

### OPEN RESEARCH BADGES

This article has been awarded Open Materials, Open Data, Preregistered Research Designs Badges. All materials and data are publicly accessible via the Open Science Framework at http://digital.csic.es/, http://hdl.handle.net/10261/214072, http://hdl.handle.net/10261/214073.

## Data Availability

The data from this study are available at http://digital.csic.es/ at the links http://hdl.handle.net/10261/214072 (Figure 1) and http://hdl.handle.net/10261/214073 (Figures 2 and 3).
